# Supplementary material for: Modification of Breakfast Fat Composition Can Modulate Cytokine and Other Inflammatory Mediators in Women: A Randomized Crossover Trial
Source: Nutrients. 2023 Aug 24;15(17):3711. doi: 10.3390/nu15173711 (PMC10489665; doi:10.3390/nu15173711)
Supplement: Supplementary file 1 [file nutrients-15-03711-s001.zip › nutrients-2554796-supplementary.pdf]

Table S1. Nutrient analysis and fatty acid composition of the different types of breakfast (based on the manufacturer's information).

	MARGARINE	BUTTER	VIRGIN OLIVE OIL
**Energy (Kcal)**	445,84	448,84	479,84
**Carbohydrates (g)**	54,61	54,53	54,43
**Proteins (g)**	16,34	16,30	16,16
**Dietary fiber**	6,35	6,35	6,36
**Fatty acid composition**			
**SFA**	22.09	66.46	15.65
Butyric acid (4:0)	-	3.95	-
Caproic acid (6:0)	-	2.47	-
Caprylic acid (8:0)		1.49	-
Capric acid (10:0)	0.17	3.09	-
Lauric acid (12:0)	2.84	3.21	-
Myristic acid (14:0)	1.00	9.14	-
Palmitic acid (16:0)	10.75	29.79	15.55
Stearic acid (18:0)	7.33	13.32	0.10
**MUFA**	26.75	27.93	72.72
Palmitoleic acid (16:1n-9)	-	1.86	-
Oleic acid (18:1n-9)	26.75	26.07	72.72
**PUFA**	50.17	4.71	11.11
Linoleic acid(18:2n-6)	49.17	4.34	10.20
α-linolenic acid (18:3n-3)	1.00	0.37	0.91
Cholesterol (mg)	-	215	-

Fatty acids data represents % fatty acids excepting cholesterol, which indicates mg/100g food. SFA: saturated fatty acids; MUFA: monounsaturated fatty acids; PUFA: polyunsaturated fatty acids.

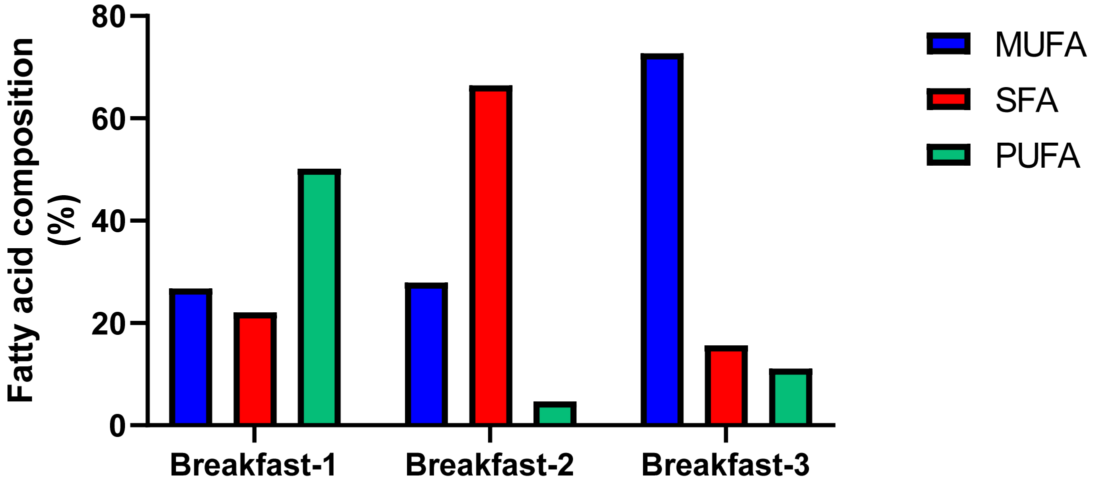

Figure S1. Fatty acid composition (in fat grams per serving) of the different interventions (based on the manufacturer's information). Breakfast-1 was mainly composed by PUFA, breakfast-2 by SFA and breakfast-3 by MUFA (the figure has been described elsewhere [1]).

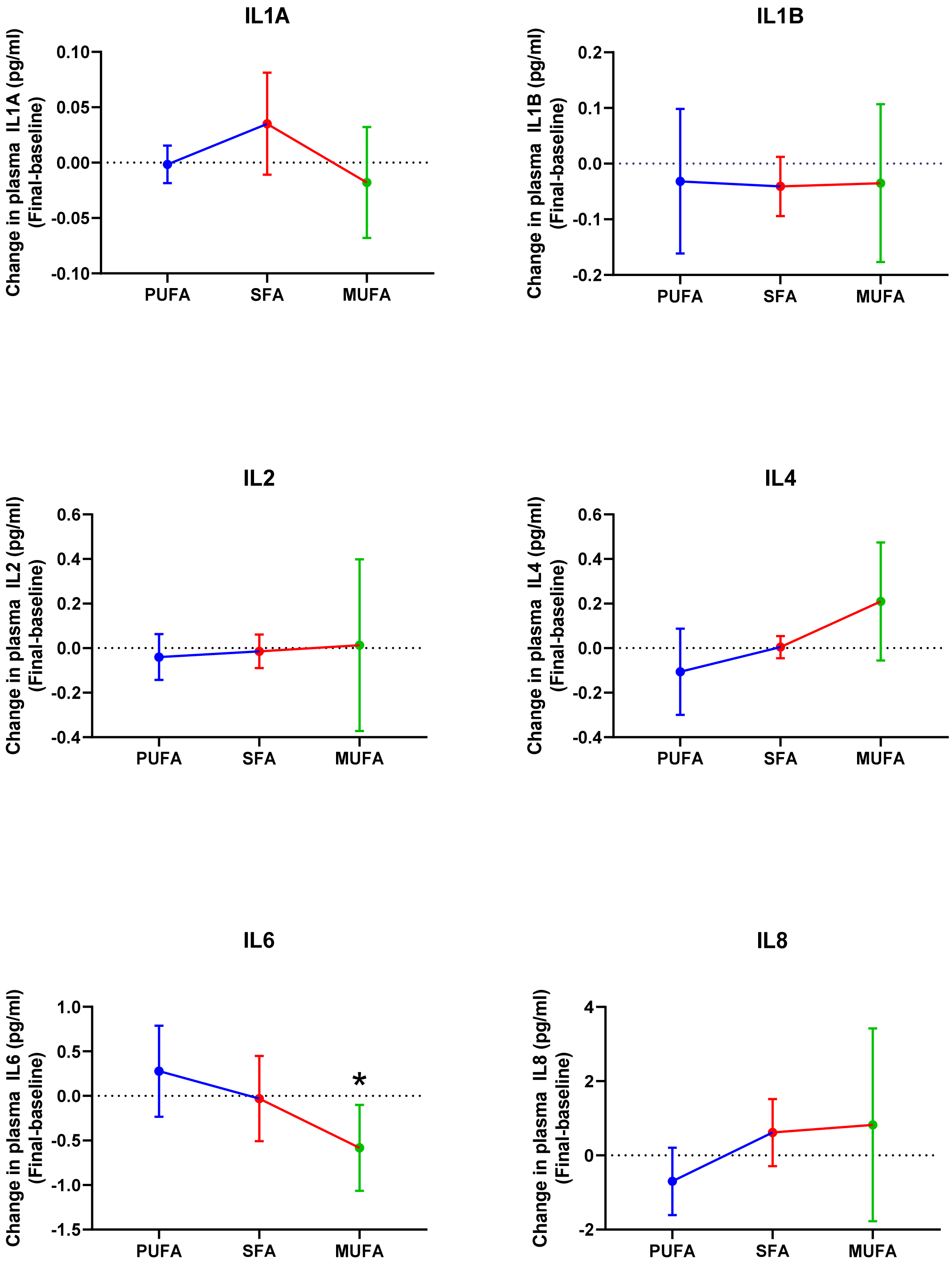
IL1A
Change in plasma IL1A (pg/ml)
(Final-baseline)
0.10
0.05
0.00
-0.05
-0.10
PUFA
SFA
MUFA
IL1B
Change in plasma IL1B (pg/ml)
(Final-baseline)
0.2
0.1
0.0
-0.1
-0.2
PUFA
SFA
MUFA
IL2
Change in plasma IL2 (pg/ml)
(Final-baseline)
0.6
0.4
0.2
0.0
-0.2
-0.4
PUFA
SFA
MUFA
IL4
Change in plasma IL4 (pg/ml)
(Final-baseline)
0.6
0.4
0.2
0.0
-0.2
-0.4
PUFA
SFA
MUFA
IL6
Change in plasma IL6 (pg/ml)
(Final-baseline)
1.0
0.5
0.0
-0.5
-1.0
-1.5
*
PUFA
SFA
MUFA
IL8
Change in plasma IL8 (pg/ml)
(Final-baseline)
4
2
0
-2
PUFA
SFA
MUFA

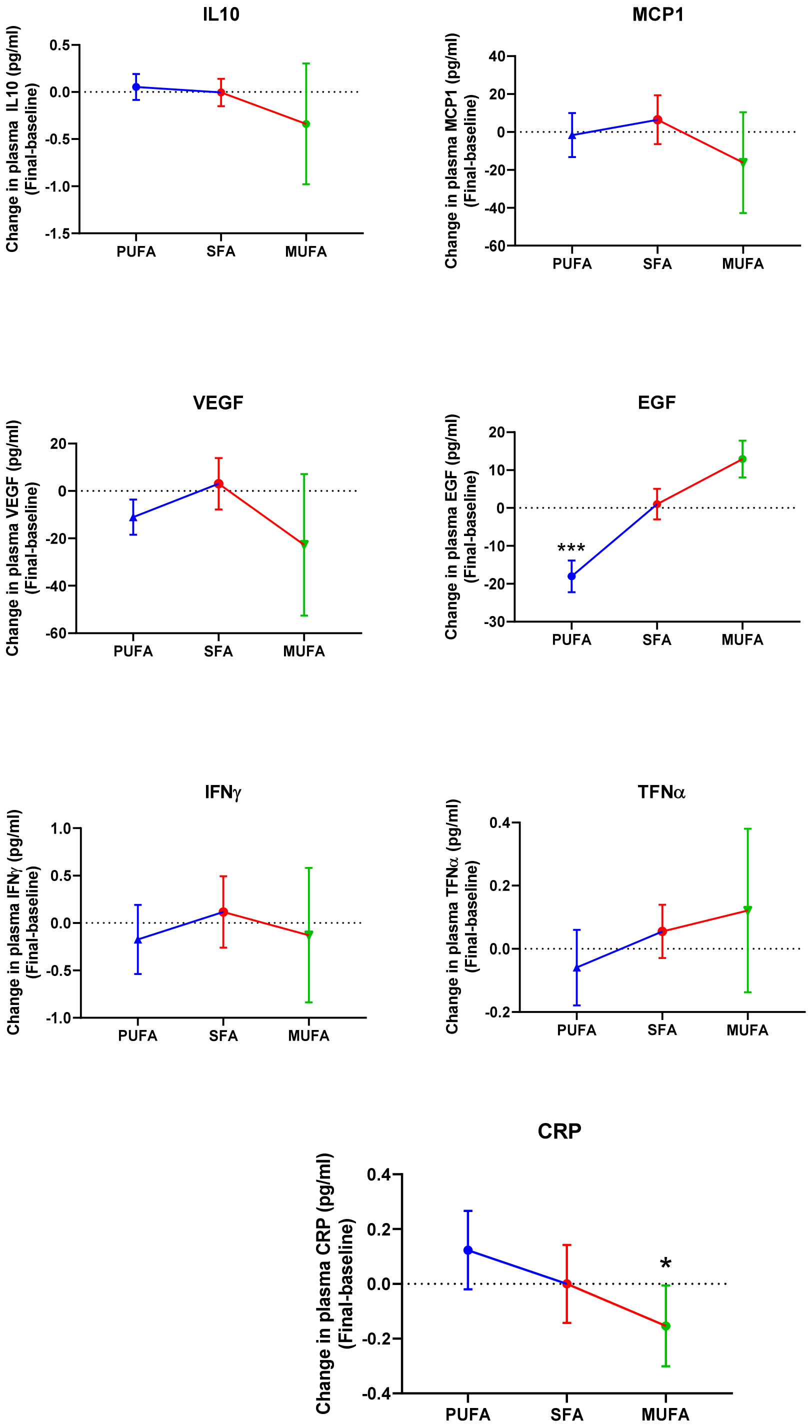

Figure S2. Estimated treatment differences (final – baseline) in all the inflammatory markers analysed in the present study. Data derived from those women who completed the study (n=51). Repeated-measures ANCOVA was employed to evaluate possible statistical differences. Baseline age, BMI and intervention order were used as covariates. PUFA represents margarine-based breakfast. SFA represents butter-based breakfast. MUFA represents extra virgin olive oil (VOO) breakfast. *Statistically significant differences between VOO-breakfast and margarine breakfast (p=0.025). *** Statistically significant differences between margarine and VOO breakfasts (p<0.001).

REFERENCES

1. Delgado-Alarcón, J.M.; Morante, J.J.H.; Aviles, F. V.; Albaladejo-Otón, M.D.; Morillas-Ruíz, J.M. Effect of the Fat Eaten at Breakfast on Lipid Metabolism: A Crossover Trial in Women with Cardiovascular Risk. *Nutrients* **2020**, *12*, 1–13, doi:10.3390/nu12061695.
